# Supplementary material for: Mild SARS-CoV-2 infection in rhesus macaques is associated with viral control prior to antigen-specific T cell responses in tissues
Source: Sci Immunol. 2022 Mar 10:eabo0535. doi: 10.1126/sciimmunol.abo0535 (PMC8995035; doi:10.1126/sciimmunol.abo0535)
Supplement: Supplementary file 1 — Figs. S1 to S6 Tables S1 and S2 [file sciimmunol.abo0535_sm.pdf]

Science Immunology
AAAS

Supplementary Materials for

Mild SARS-CoV-2 infection in rhesus macaques is associated with viral control prior to antigen-specific T cell responses in tissues

Christine E. Nelson *et al.*

Corresponding author: Daniel L. Barber, barberd@niaid.nih.gov

DOI: 10.1126/sciimmunol.abo0535

The PDF file includes:

Figs. S1 to S6
Tables S1 and S2

Other Supplementary Material for this manuscript includes the following:

Data file S1

Supplementary Materials:

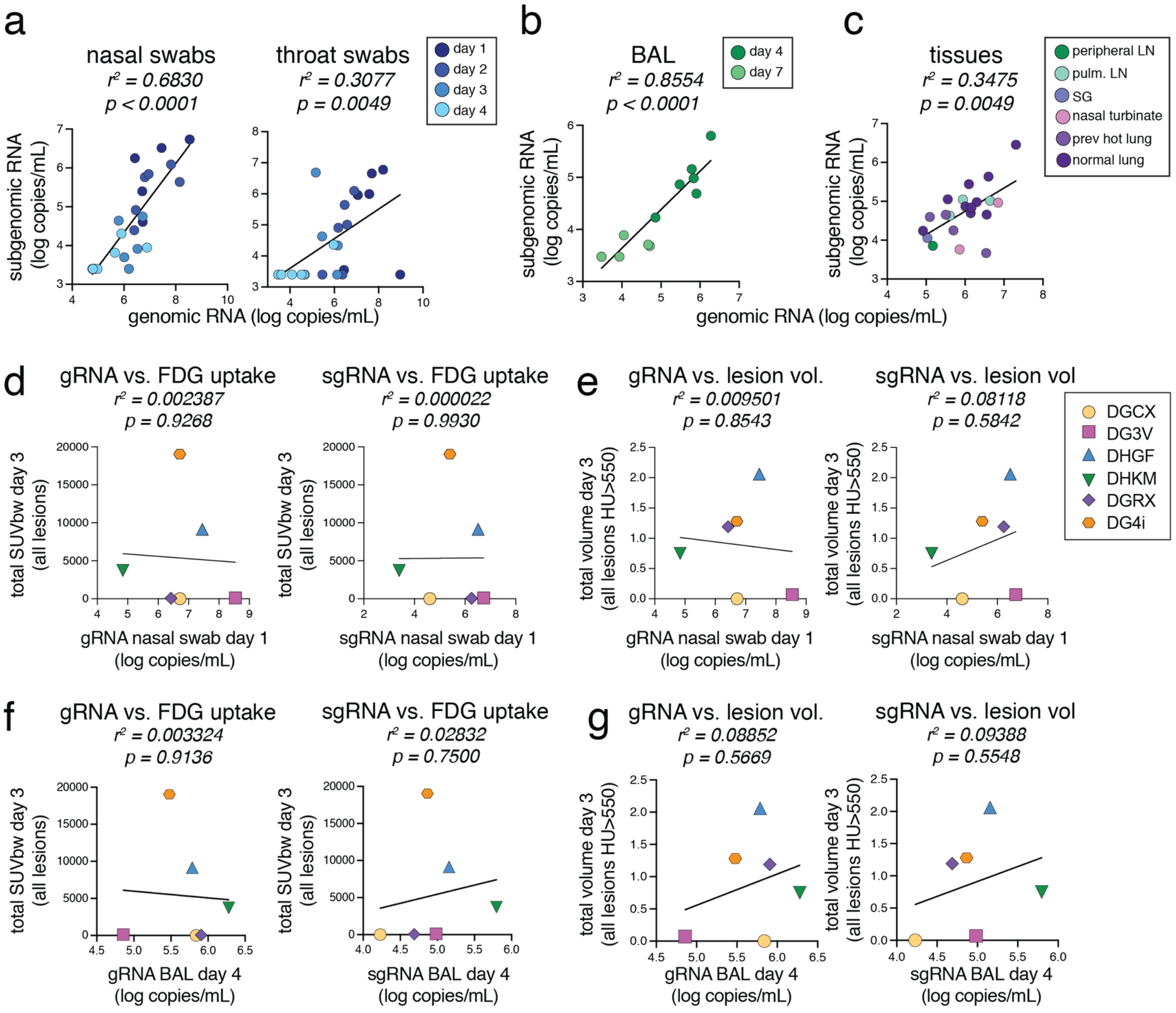

Figure S1. Viral RNA levels are not correlated with disease severity. Correlation analysis of viral RNA and lung lesion severity. (A) Correlation of genomic RNA and subgenomic RNA in copies/mL in nasal swabs (left graph) and throat swabs (right graph) from day 1 to day 4, as shown in figure 1D. (B) Correlation of genomic RNA and subgenomic RNA in copies/mL in BAL from day 4 and day 7, as shown in figure 1D. (C) Correlation of genomic RNA and subgenomic RNA in copies/gram of tissue from day 10 in tissues with values > limit of detection, as shown in figure 1E. (D) Correlation of genomic RNA (left graph) or subgenomic RNA (right graph) at day 1 in nasal swabs and the sum of lung lesion metabolic activity at day 3. (E) Correlation of genomic

RNA (left graph) or subgenomic RNA (right graph) at day 1 in the nasal swabs and the sum of lung lesion size at day 3. (F) Correlation of genomic RNA (left graph) or subgenomic RNA (right graph) at day 4 in the BAL and the sum of lung lesion metabolic activity at day 3. (G) Correlation of genomic RNA (left graph) or subgenomic RNA (right graph) at day 4 in the BAL and the sum of lung lesion size at day 3.

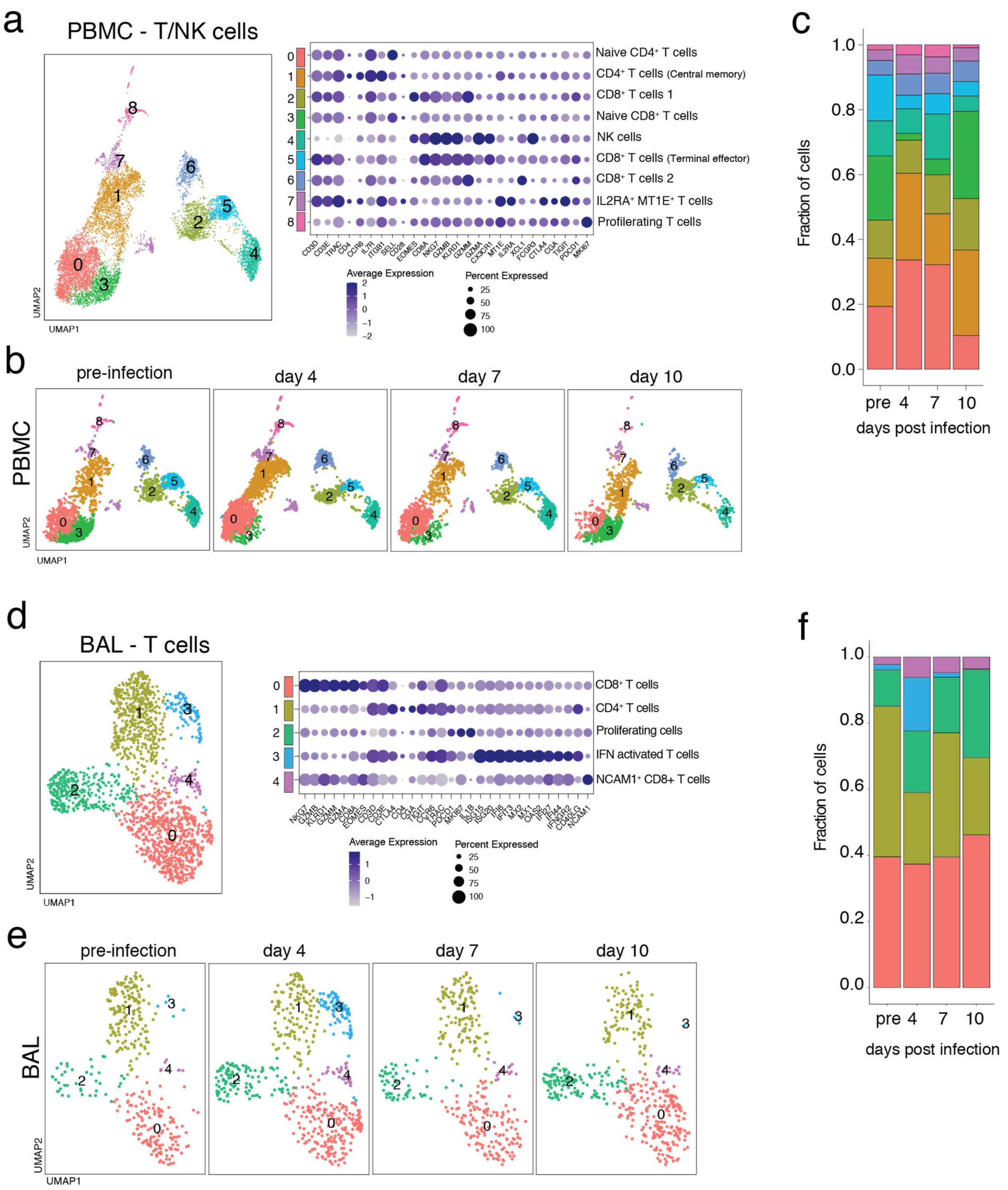

Figure S2. Minor alterations in T cells and NK cells in PBMC and BAL as measured by scRNAseq. (A, D) UMAP plot shows the sub-clustering of PBMC T and NK cells (A) from Figure 2A and BAL T cells (D) from Figure 3A (top panels). Clusters were annotated with cell-types based

on gene expression patterns as shown on the dot plot and are identified with different numbers and colors on the plots. (lower panels). (B, E) UMAP plots depict the kinetic of lymphoid cells over time. (C, F) Fraction of cells present in each of the lymphoid cell clusters in PMBC (C) and BAL (F) is summarized.

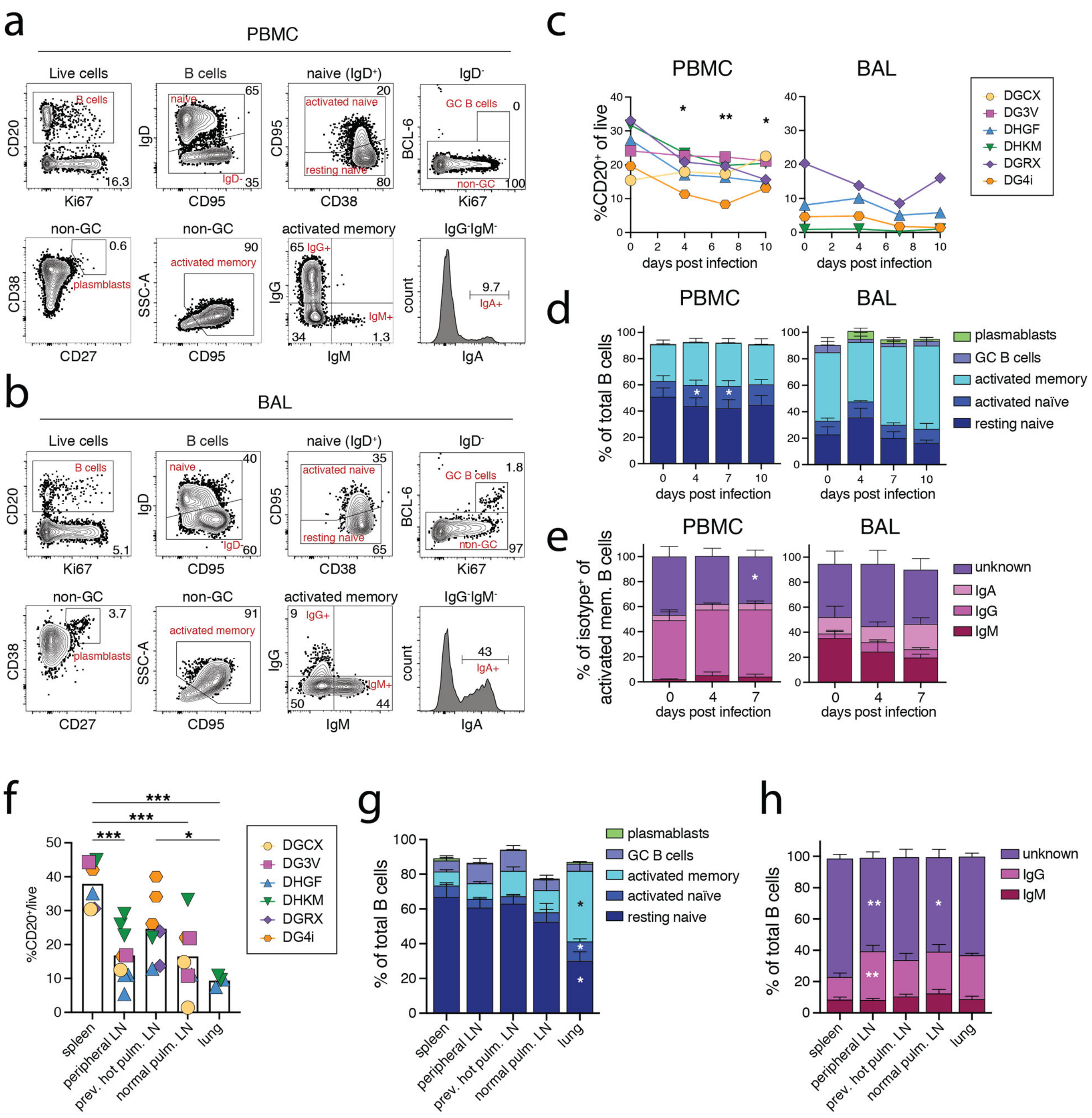

Figure S3. B cell responses after SARS-CoV-2 infection in rhesus macaques. Quantification of different B cell subsets after SARS-CoV-2 infection. Representative flow cytometry gating strategy of B cells and B cell subsets (i.e., resting naïve, activated naïve, germinal center B cells (GC B cells), plasmablasts, activated memory and different isotypes IgG$^+$, IgM$^+$, and IgA$^+$) from the blood (A) and BAL (B) from ID#DHGF at day 7. (C) Quantification of total B cells from the blood and BAL over time. Dunnett's multiple comparison test comparing to day 0. (D)

Quantification of different B cell subsets in the blood and BAL as a frequency of total B cells. Showing the mean value from all animals with standard error. Dunnett's multiple comparison test comparing values to day 0. No BAL data on B cells was collected for DGCX and DG3V. (E) Quantification of the frequency of IgG^+, IgM^+, IgA^+, and isotype undefined of activated memory B cells. Dunnett's multiple comparison test comparing values to day 0. DGCX and DG3V did not have baseline values. (F-H) Frequency of total B cells (F), subsets (G), and isotypes (H) from spleen, peripheral lymph nodes (axillary, inguinal, and/or cervical), previously hot pulmonary lymph nodes, normal pulmonary lymph nodes, and lung sections at day 10 post infection. No data from the lungs of DGCX, DG3V, DGRX, and DG4i; previously hot pulmonary lymph nodes from DGCX and DG3V; normal pulmonary lymph nodes from DGRX; or peripheral lymph nodes from DGRX. Significance for G and H done using a Turkey's multiple comparison test. In G, significant difference in lung activated memory subset vs. spleen, peripheral LN, previously hot and normal pulmonary LN. Significant difference in activated naïve in lung vs. previously hot pulmonary LN. Significant difference in resting naïve in lung vs. spleen, peripheral LN, and previously hot pulmonary LN. In H, significant difference in IgG^+ and unknown isotype in peripheral LN vs. spleen. Significant difference in unknown isotype in normal pulmonary LN vs. spleen. IgA isotype not quantified at day 10 necropsy. For all statistical analysis $p<0.05$ for the given test is considered significant: * $p<0.05$, ** $p<0.01$, *** $p<0.001$, **** $p<0.0001$.

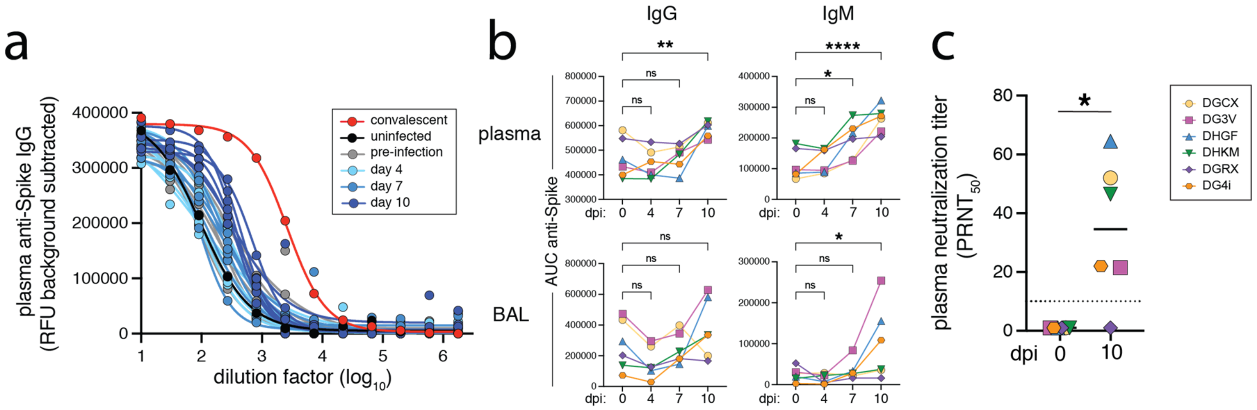

Figure S4. Antibody responses after SARS-CoV-2 infection in rhesus macaques. (A) Anti-Spike IgG antibody titration curves from plasma at pre-infection, day 4, 7, and 10 post-infection compared to uninfected and convalescent NHP plasma as measured by fluorescent ELISA. Points are relative fluorescence units (RFU) with background subtraction (value minus bottom of the interpolation curve). Lines are sigmoidal log x interpolation curves. (B) Area under the curve (AUC) of the antibody titration curves for anti-Spike IgG and IgM responses from plasma and BAL from pre-infection, days 4, 7, and 10 post-infection with background subtraction and for BAL, normalization to total protein concentration. Significance calculated with Dunnetts's multiple comparison test. (C) Live-virus plasma neutralization titers from pre-infection and day 10 post-infection, reported as $PRNT_{50}$. Data are plotted as the average of two technical replicates. The limit of detection of 10 $PRNT_{50}$ is represented as a dotted line Significance calculated with paired t-test. $p<0.05$ is considered significant: * $p<0.05$, ** $p<0.01$, *** $p<0.001$, **** $p<0.0001$.

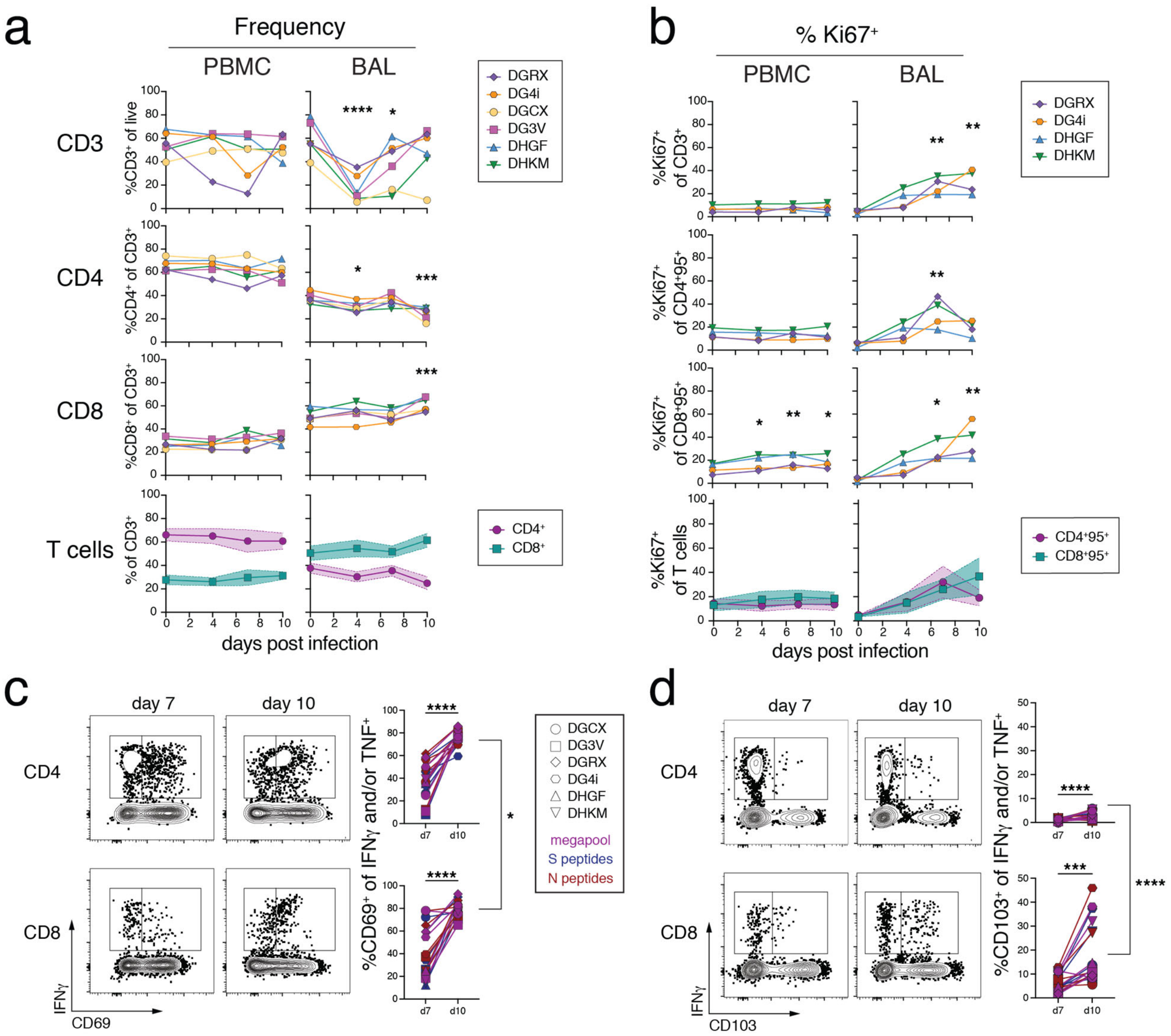

Figure S5. Activation of bulk and antigen-specific T cell responses in the blood and BAL. (A) Frequency of bulk CD3+, CD4+ or CD8+ responses overtime in the blood and BAL. Significance indicated with Dunnett's multiple comparison test comparing individual timepoint to baseline. Bottom set of graphs overlay the mean CD4+ and CD8+ frequency of CD3+, response with standard deviation. (B) Frequency of Ki67+ on bulk CD3+, CD4+CD95+, or CD8+CD95+ in the blood and BAL. Bottom set of graphs overlay the mean Ki67+ on CD4+CD95+ and CD8+CD95+ response with standard deviation. Ki67 stain not done in DGCX or DG3V. (C) Representative flow cytometry plots from ID#DG4i and quantification of the frequency of CD69+ on Ag-specific CD4 and CD8 T cells from the BAL on day 7 and day 10 post infection. Paired t-test comparing day 7 vs. day 10

for CD4 and CD8 separately, and CD4 day 10 vs. CD8 day 10. (D) Representative flow cytometry plots from ID#DG4i and quantification of the frequency of $CD103^{+}$ on Ag-specific CD4 and CD8 T cells from the BAL on day 7 and day 10 post infection. Paired t-test comparing day 7 vs. day 10 for CD4 and CD8 separately, and CD4 day 10 vs. CD8 day 10. For C and D only samples with >35 data points were included for phenotypic analysis. For all statistical analysis $p<0.05$ for the given test is considered significant: * $p<0.05$, ** $p<0.01$, *** $p<0.001$, **** $p<0.0001$

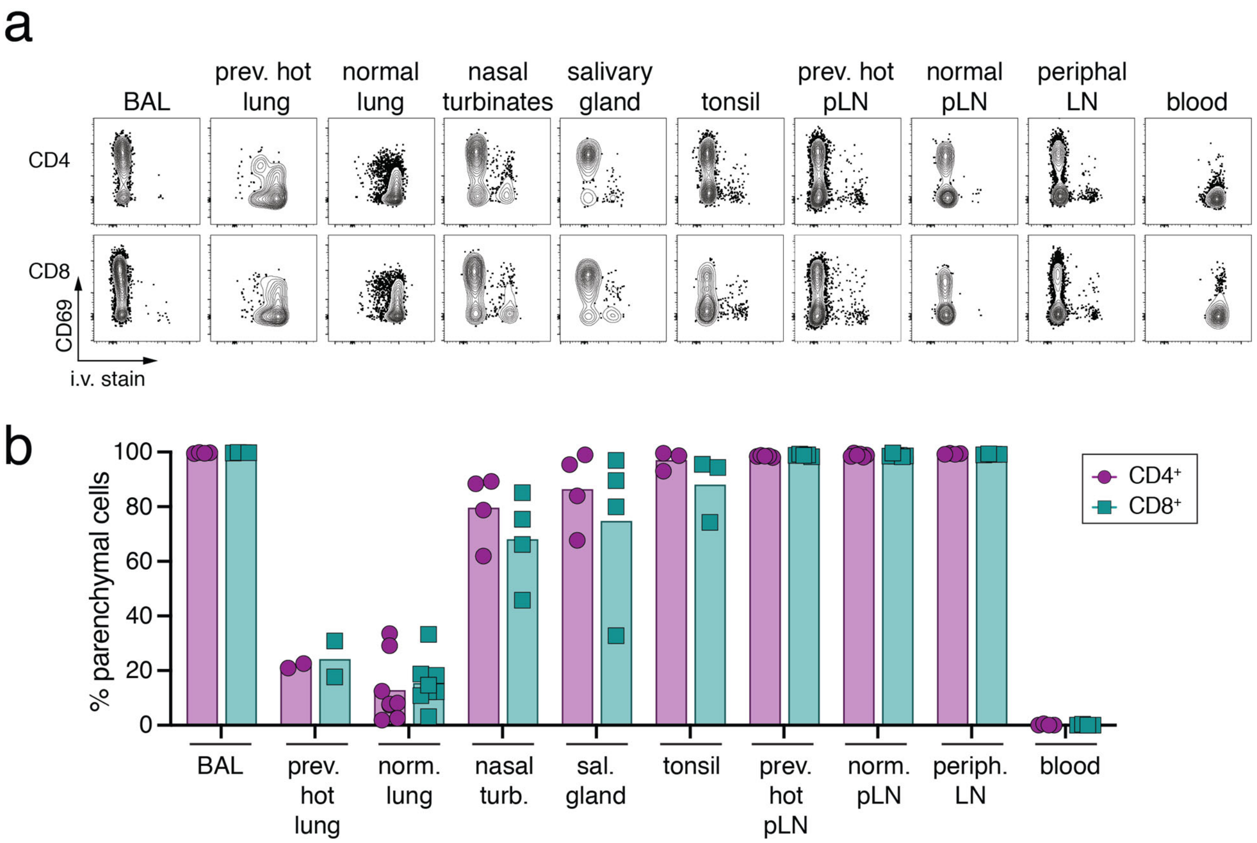

Figure S6. Parenchymal localization of T cells in tissues from SARS-CoV-2 rhesus macaques. To determine tissue parenchymal localization of T cells, animals were injected with an anti-CD45-biotin antibody 10 minutes prior to exsanguination and necropsy. Intravenous antibody stain (i.v. stain) positive cells are localized to the tissue vasculature, while i.v. stain negative cells are localized to the tissue parenchyma (See Methods). (A) Representative flow cytometry plots of i.v. stain vs. CD69 on $CD4^+CD95^+$ and $CD8^+CD95^+$ from BAL, previously PET hot lung, normal lung, nasal turbinates, salivary gland, tonsil, previously hot pulmonary LN (pLN), normal pLN, peripheral lymph nodes (axillary), and blood from ID#DGRX at necropsy, except salivary gland, which was from ID#DGCX. (B) Quantification of parenchymal $CD4^+CD95^+$ and $CD8^+CD95^+$ T cells (equivalent to i.v. stain negative) from tissues at necropsy. DHGF and DHKM did not have i.v. stain.

Table S1: Study animal information.

animal ID	sex	Age years	weight kg	experimental round
DGCX	Male	5.58	7.86	1
DG3V	Male	4.58	6.81	1
DHGF	Male	2.58	3.7	2
DHKM	Male	2.92	3.96	2
DGRX	Male	4.83	9.68	3
DG4i	Male	4.83	10.01	3

Table S2: Monoclonal antibody information.

Flow Cytometry antibodies

antibody	clone	manufacturer
CD69	FN50	Biolegend
Granzyme B	GB11	BD
CD8a	RPA-T8	Biolegend
IL-2	MQ1-17H12	Biolegend
IFNg	4S.B3	Biolegend
IL-17A	FN50	Biolegend
TNFa	Mab11	BD
CD4	SK3	BD
CD95	DX2	BD
CD3	SP34-2	BD
CD107a	H4A3	Biolegend
CD107b	H4B4	Biolegend
ViabilityDye eFluoro780		Thermo
CD103	B-Ly7	ebioscience
CD28	CD28.2	Biolegend
Ki67	JES3-9D7	BD
IgD	AB_2795624	Southern Biotech
BCL-6	K112-91	BD
IgM	G20-127	BD
HLA-DR	L243	Biolegend
CD95	Dx2	Biolegend
CD20	2H7	Biolegend
CXCR3	1C6/CXCR3	BD
CCR6	11A9	BD
CD27	L128	BD
IgA	A9604D2	Southern Biotech
Streptavidin		BD
IgG	G18-145	BD
CD38	OKT10	CapricoBio
Ki-67	B56	BD

TotalSeq hashtag antibodies

antibody	clone	manufacturer
TotalSeq™-A0251	LNH-94; 2M2	Biolegend
TotalSeq™-A0252	LNH-94; 2M2	Biolegend
TotalSeq™-A0253	LNH-94; 2M2	Biolegend
TotalSeq™-A0254	LNH-94; 2M2	Biolegend
TotalSeq™-A0255	LNH-94; 2M2	Biolegend
TotalSeq™-A0256	LNH-94; 2M2	Biolegend
TotalSeq™-A0257	LNH-94; 2M2	Biolegend
TotalSeq™-A0258	LNH-94; 2M2	Biolegend
TotalSeq™-A0259	LNH-94; 2M2	Biolegend
TotalSeq™-A0260	LNH-94; 2M2	Biolegend
TotalSeq™-A0262	LNH-94; 2M2	Biolegend
TotalSeq™-A0263	LNH-94; 2M2	Biolegend
TotalSeq™-A0264	LNH-94; 2M2	Biolegend
TotalSeq™-A0265	LNH-94; 2M2	Biolegend
